# Fixational eye movements in passive versus active sustained fixation tasks

**DOI:** 10.1167/jov.21.11.16

**Published:** 2021-10-22

**Authors:** Norick R. Bowers, Josselin Gautier, Samantha Lin, Austin Roorda

**Affiliations:** 1 Herbert Wertheim School of Optometry and Vision Science, University of California, Berkeley, CA, USA

**Keywords:** fixational eye movements, preferred retinal locus for fixation, adaptive optics, scanning laser ophthalmoscopy

## Abstract

Human fixational eye movements are so small and precise that high-speed, accurate tools are needed to fully reveal their properties and functional roles. Where the fixated image lands on the retina and how it moves for different levels of visually demanding tasks is the subject of the current study. An Adaptive Optics Scanning Laser Ophthalmoscope (AOSLO) was used to image, track and present a variety of fixation targets (Maltese cross, disk, concentric circles, Vernier and tumbling-E letter) to healthy subjects. During these different passive (static) or active (discriminating) tasks under natural eye motion, the landing position of the target on the retina was tracked in space and time over the retinal image directly with high spatial (<1 arcmin) and temporal (960 Hz) resolution. We computed both the eye motion and the exact trajectory of the fixated target's motion over the retina. We confirmed that compared to passive tasks, active tasks elicited a partial inhibition of microsaccades, leading to longer drift periods compensated by larger corrective saccades. Consequently, the overall fixation stability during active tasks was on average 57% larger than during passive tasks. The preferred retinal locus of fixation was the same for each task and did not coincide with the location of the peak cone density.

## Introduction

When fixating our gaze on an object, our eyes are never truly at rest. Even while staring at a small object, like the bottom row of a Snellen acuity chart, our eyes are constantly in motion. Small, fast microsaccades and slow drifts constantly shift the image of the fixation target over the photoreceptor lattice. These fixational eye movements (FEM) are a nuisance for many ophthalmic measurements such as imaging, microperimetry or retinal and refractive surgery. However, owing to the fact that FEM are the finest motor control system in the human body, they offer an opportunity for early detection and monitoring of neurological disorders (Hunfalvay et al., 2021; Montesano et al., 2018; Sheehy et al., 2020). It is also increasingly clear that these small movements are not simply noise in the oculomotor system (Rucci & Victor, 2015), but that they serve a number of important functions in the visual system, such as preventing fading (Martinez-Conde et al., 2006). Microsaccades have been found to be associated with shifts in attention (Engbert & Kliegl, 2003; Hafed & Clark, 2002), and fine scale repositioning stimuli within the foveola (Intoy & Rucci, 2020; Ko et al., 2010; Poletti et al., 2013). Whereas drift has been shown to enhance the discrimination of fine spatial details through a combination of spatiotemporal enhancement at high frequencies (i.e., whitening of the power spectrum) (Rucci, 2008; Rucci et al., 2007) and enrichment of the information relayed from the retina to the brain through dynamic sampling (Ahissar & Arieli, 2012; Anderson et al., 2020; Burak et al., 2010; Ratnam et al., 2017).

A major limitation in studying the smallest FEM is the instrument used to measure the gaze itself (Poletti & Rucci, 2016). Modern video-based eye trackers are convenient but often lack the resolution of earlier systems. Scleral search coils, which were among the first high-resolution eye trackers, have high temporal and spatial resolution (Collewijn et al., 1975; Robinson, 1963) but are invasive and difficult to use. Dual Purkinje Image (DPI) eye trackers came to use shortly after search coils and offered a noninvasive way to attain high accuracy for tracking the gaze (Crane & Steele, 1985) and still represent a reliable eye tracker today (Fourward Technologies, Gallatin, MO). However, the DPI system has been shown to have its own drawbacks. The crystalline lens, which is the source of the fourth Purkinje image that is tracked in a DPI system, can move independently from the rest of the eyeball giving rise to spurious measurements of gaze direction especially for tremor or postsaccadic overshoot (Bowers et al., 2019; He et al., 2010; Nyström et al., 2015; Tabernero & Artal, 2014). On the other hand, modern video eyetrackers, today's most commonly used instruments in research and industry, suffer from pupil size changes (Choe et al., 2016; Hooge et al., 2016; Nyström et al., 2016). They also simply lack the requisite spatial resolution to accurately estimate the gaze position produced by these FEMs (Holmqvist & Blignaut, 2020; Kimmel et al., 2012).

FEM have drawn renewed interest in research lately as the field progresses toward higher and higher resolution structural and functional measurements of the retina and as the role of FEM in fine-scale vision continues to be examined. The effects of fixation target and task have not been thoroughly examined using high resolution retinal-image–based tracking techniques. Published studies, which primarily relied on video eyetrackers, generally showed that smaller targets elicit modestly less overall FEM compared with larger targets (Kazunori et al., 2016; McCamy et al., 2013). Other target properties, such as shape, color, contrast, blur and luminance in eliciting improved fixation stability have been more scarce (Bhattarai et al., 2019; Steinman 1965; Thaler et al., 2013; Ukwade & Bedell, 1993) with few trends emerging except that a bull's eye and cross targets (or a combination of both) elicit the least FEM (Thaler et al., 2013). Most experiments that require fixation use a simple static fixation target (see Thaler, 2013 for a comprehensive overview of the variety of targets commonly used).

The implication of FEM in encoding visual information (McCamy et al., 2014; Otero-Millan et al., 2008), as well as their modulation during fine discrimination tasks (Rucci & Victor, 2015) is well-documented. The variations in subjects’ stability over different kinds of controlled tasks is less understood. A more comprehensive characterization of FEM and fixation target is therefore important for several reasons. A high resolution set of unambiguous oculomotor data reporting intra- and inter-individual variability during various tasks offers an important baseline for improved interpretation of FEM in health and disease as well as to better understand their functional roles. Finally, a more practical reason is simply to learn what target and/or fixation task might minimize overall FEM in clinical settings where motion and its consequent blurred or distorted retinal images can be detrimental, such as with fundus photography, optical coherence tomography (OCT) scans, or fundus-guided microperimetry.

The current study aims to compare and contrast FEM during *active* tasks–those that contain temporal variation and require subject input—and *passive* tasks, where the subject is simply instructed to maintain fixation on a target. An adaptive optics scanning laser ophthalmoscope (AOSLO) is used as an eye tracker to acquire high spatial (<1 arcmin) and temporal (960 Hz) resolution eye traces. Because the AOSLO can also obtain an unambiguous record of the motion of the target that is projected onto the retinal surface, we compare how the preferred retinal locus for fixation (PRL) relates to the location of peak cone density (PCD) for each type of fixation target.

## Methods

Eight healthy subjects (self-reported), three male and five female, with normal or corrected-to-normal vision participated in the experiment. Subject ages ranged from 23 to 53 years old. All experimental procedures adhered to the conditions set by the institutional review board of the University of California, Berkeley, and followed the tenets of the Declaration of Helsinki. Each subject read and signed a written informed consent document. Before imaging, the subjects’ eyes were dilated and cyclopleged using 1 drop each of 1% tropicamide and 2.5% phenylephrine. The drops were used to provide maximum dilation for imaging as well as to paralyze accommodation, both of which help to ensure high quality images in the AOSLO. No detectable difference has been found between eye traces measured in an SLO system with or without dilation (Bowers et al., 2019).

### AOSLO system

Data were recorded using the AOSLO (Roorda et al., 2002), which is used to image and track the retina as well as to provide the fixation targets used in this experiment. For imaging, a point source of light is relayed through the optical path and scanned across the retina in a raster pattern utilizing two scanners, a 16 kHz fast horizontal scan and a 30 Hz slow vertical scan. The reflected light is descanned through the optical path and directed to a custom-built Shack–Hartmann wavefront sensor and through a confocal pinhole to a photomultiplier tube (Hamamatsu, Japan). The Shack–Hartmann wavefront sensor is used to measure the optical aberrations and send a correction to the deformable mirror (7.2 mm diameter, 97 actuators membrane; ALPAO, Montbonnot-Saint-Martin, France) in the optical path. Light detected by the PMT and the positional information from the scanner are combined to construct videos of the retina with 512 × 512 pixel sampling resolution at a frame rate of 30 Hz (the speed of the slow vertical scanner). In this experiment, the eye's pupil was kept in a fixed position relative to the AOSLO beam by restraining the subject's head movement through the use of a dental bite bar and temple mounts. The nonimaged eye was covered with an eye-patch. The imaging wavelength was 680 nm, with 940 nm used for wavefront sensing. The field size of the video was 0.9 × 0.9${}^{\circ }$. Using an average power of 50 to 70 µW, the raster scan field appeared as a bright red square flickering at a rate of 30 Hz to the subject. Fixation targets were presented to the subject within the red field by turning off the scanning laser using an acousto-optic modulator (Brimrose Corp, MD) at the appropriate time points during the raster scan. To the subject, these targets appeared as black-on-red decrements. The stimuli were very sharp and had high contrast owing to the use of adaptive optics on the input scanning beam. Importantly, these decrements are also encoded directly into the video, which allows for an unambiguous measurement of the motion of the image of the fixation target over the retina. This system is capable of obtaining near diffraction-limited images of the photoreceptor mosaic and delivering stimuli with the precision of 1 pixel (approximately 6 arcseconds). An example video from one of the concentric circle trials in this experiment is shown in the Supplementary Materials. This system has been explained in greater detail in previous manuscripts from our group (Poonja et al., 2005; Rossi & Roorda, 2010).

### Experiment design

The experiment consisted of five different conditions: Maltese cross, disk, concentric circles, Vernier acuity, and a tumbling E (M, D, C, V, and E, respectively). The Maltese cross condition (M) was chosen as it has been suggested to provide a better fixation target than the simple dot that is commonly used in fixation tasks. The disk condition (D) consisted of an annulus within the center of the raster that the subjects were instructed to fixate. Both of these conditions were simple fixation tasks where subjects were instructed to hold their gaze on the target. The concentric circles condition (C) consisted of concentric rings moving in a constricting radial motion. There were six rings ranging in size from 10 to 1 arcmin that were presented over the course of 18 frames (three frames per ring size) and replayed every 30 frames for a frequency of 1 Hz. The aim of the concentric rings was to provide a fixation task that was similar to the passive task in that it required no subject response, but was also similar to the active task in that it was dynamically changing. As such, it provided a control to distinguish whether any FEM differences could be attributed to the active task or due to the fact that the stimulus was dynamic. The Vernier hyperacuity condition (V) required subjects to judge the relative displacement of two horizontal bars which appeared at random intervals (seven 6-arcsecond steps). The tumbling E condition (E) consisted of a tumbling E task where the subjects were asked to report the orientation of a letter E as it rotated randomly. The size of the E varied in seven steps, from 20/6 to 20/20 Snellen acuity. For both V and E tasks the stimulus was presented for 0.5 seconds (15 consecutive frames) and there were random time intervals between presentations — evenly spread over 0.5 to 1.5 seconds — where nothing was presented. The random time intervals were used so that subjects could not anticipate the next trial and were therefore compelled to maintain fixation the entire time. The V and E condition can be differentiated from the others because they both required subject judgment and response, as well as providing temporal variation. These conditions were further categorized into passive tasks (M, D) and active tasks (E, V) for analysis depending on whether they required subject response and varied in time. The different fixation targets were presented in a pseudo-random order to eliminate any training or fatigue effects. Furthermore, subjects were given consistent instructions from a script to avoid known changes in behavior due to instruction (Steinman et al., 1967). The full script is provided in the Supplementary Materials but the primary emphasis in the instruction was for the subject to maintain their gaze throughout the entire duration of each 36-second trial task. There were five 36-second trials for each condition in total.

**Table 1.** Illustration of the 5 experimental conditions and their respective parameters. The different colors indicate distinctions between passive (green), active (purple), and mixed (orange) tasks. This color scheme will be used throughout to differentiate the 5 conditions. *Snellen fraction

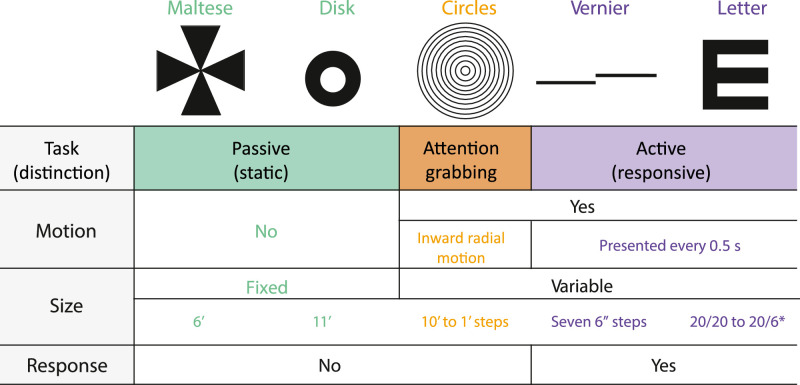

### Eye tracking and video processing

Because this system uses a raster scanning technique (i.e., each frame is acquired over time), eye motion information is available beyond the 30 Hz frame rate. This information can be extracted to achieve eye traces at temporal resolution many times greater than the frame rate of the movies (Stevenson & Roorda, 2005; Vogel et al., 2006). To acquire eye traces at higher temporal resolution than the 30 Hz frame rate, each frame of the AOSLO movie is broken into horizontal strips and cross-correlated against a reference frame. This analysis is done offline using custom software written in Matlab (The Mathworks, Natick, MA) (Stevenson et al., 2010). This technique allows collection of eye traces at high spatial (<1 arcmin) and temporal (960 Hz) resolution. Eye traces were separated into drifts and saccades using a semi-automatic software and the output was manually verified by the authors. Saccade onset was defined as the point when instantaneous speed exceeded 1.5 °/sec and offset was defined as the point when the trace fell back below this threshold. Blinks were defined as frames of the AOSLO movie when the mean luminance fell below a threshold that was defined on a per-subject basis dependent on the average brightness of the respective movies.

The AOSLO records high-resolution videos of the retina for each trial and the fixation target is directly encoded into the video, thereby making it possible to plot the exact path of the fixation target over the photoreceptor mosaic directly. This is done in the following way. First, the eye motion traces extracted from AOSLO videos indicate how the entire retina moves, but do not directly indicate where the fixation target lands on the retina. Computing the actual retinal trajectory requires computing the ΔX and ΔY offsets that need to be applied to each eye trace to anchor it to the exact position of the fixation target on the retina. To accomplish this step, we first generate a high-quality master retinal image chosen from one of the best videos recorded in the experimental session for each subject. Then we use the same cross correlation methods to align strips containing the encoded stimulus from each video with that master retinal image and determine the position of the stimulus on the master retinal image. The X–Y position corresponding to the strip that contains the stimulus is then aligned to that exact position on the master retinal image using these ΔX and ΔY offsets. In theory, the offset only needs to be computed once for a single strip, but the match between a single strip and the master retinal image can have small errors due to noise in the strip or torsion in the retinal image. So, to improve accuracy, we compute the average offsets from at least 20 unique strips, ensuring that the standard deviation of the offsets is less than 2 pixels (0.2 arcmin). These processing steps yield accurate trajectories in retinal coordinates for every trial and every condition, all referenced to a single master retinal image.

**Figure 1.**
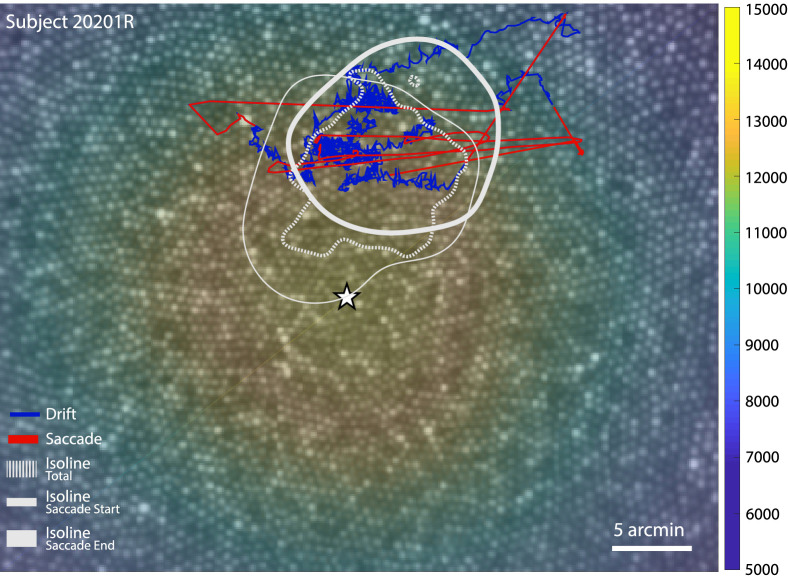
Master retinal image for subject 20201R with functional and structural measures overlaid. The star indicates the point of maximum cone density (PCD) and the underlying colormap represents the cone density in cones per degree${}^{2}$. Five seconds of eye movement is plotted on the retinal image representing the stimulus motion on the retina with saccades (red) and drifts (blue) highlighted. The isoline contours for all the eye positions obtained during the Vernier condition (dotted), as well as saccade start (thin) and end points (thick) for this condition are shown in gray (see Figures 4 and 6 for contours for all conditions and all subjects).

For all of our subjects, the master retinal image was of sufficient quality to label all cones across the image. Cones were labeled across the entire foveal region using a combination of automatic cone-finding (Li & Roorda, 2007) with manual intervention when necessary. Cone density was computed within a 10-arcmin diameter circular window while it traversed, pixel by pixel, across the mosaic (using a convolution process). The 10-arcmin averaging window was chosen since it has been shown to strike an optimal balance between minimizing noise and maximizing resolution (Wang et al., 2019). The point of maximum cone density was expressed as the pixel location with the highest density value. This analysis allows us to determine how the location on the retina the subject used to examine the stimulus (the PRL) differed from the PCD on the retinal lattice. Figure 1 shows an example of a master retinal image from one subject with selected structural and functional measures overlaid onto it.

### Eye movements, ISOA, and PRL analysis

The ISOA method was used to measure fixation stability as it was proposed as a better alternative to bivariate contour ellipse area in the presence of multiple loci of fixation positions (Castet & Crossland, 2012). It does not make any assumption on the nature of the random variables underlying the distribution of data points, which is specifically appropriate for people with eccentric fixation (Whittaker et al., 1988), but also normal subjects whose fixational eye positions have been shown to not be randomly distributed (Cherici et al., 2012). The ISOA and PRL are computed through kernel density estimation of the two-dimensional (2D) probability density function (PDF) of eye positions. The ISOA is the area within the nonuniform contour that encompasses 68% of the entire eye trace. The PRL is computed as the corresponding peak of the 2D PDF. In other words, the isoline contour encloses all the eye positions that lie within 1 standard deviation (SD) from the PRL, if we could assume normality and a unique PRL (which we observed). To assert both non-normality of the 2D distribution of eye positions and the non-separability between pairwise distributions obtained during different conditions, one-way and two-way 2D Kolmogorov–Smirnov tests were used, respectively. This implementation relies on Fasano and Franceschini's generalization (Fasano & Franceschini, 1987) for two dimensions.

## Results

### Global eye movement statistics

**Figure 2.**
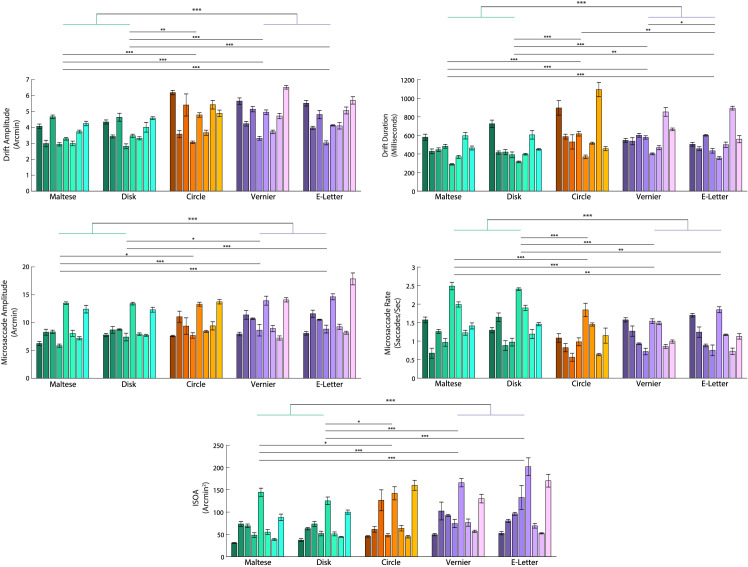
Individual subject performance across the five conditions. The passive tasks are represented by the green bars and the active tasks are represented by the purple bars. The mixed task (circles) is represented by the orange bars. Differently shaded bars indicate the mean and standard error the mean of each subject's performance across their 5 respective trials for each condition. The horizontal lines above the bar plot with asterisks represent levels of significance (*p* < 0.05, *p* < 0.01, and *p* < 0.001 respectively) from a post hoc Tukey test ran on a repeated-measures ANOVA across the five conditions for each variable. The uppermost horizontal bar with green and purple stems represents the same levels of significance from a paired-samples *t*- test but compares the two active tasks pooled together versus the two passive tasks pooled together.

Selected FEM measurements for all subjects and all conditions are plotted in Figure 2. The figure reveals expected extensive differences in FEM between subjects (Cherici et al., 2012), which is discussed elsewhere in this article. Comparing between conditions, we found that subjects had a lower saccade rate, repeated measures analysis of variance (ANOVA) F(4, 28) = 9.91, *p* < 0.001, post hoc tukey *p* < 0.05 in all passive versus active comparisons, and a higher saccade amplitude, repeated measures ANOVA F(4, 28) = 11.4 with Greenhouse–Geisser correction, *p* < 0.001, post hoc tukey *p* < 0.05 in all passive versus active comparisons, during the two active tasks compared with the two passive tasks. Correspondingly, the drift amplitude, repeated-measures ANOVA with Greenhouse–Geisser correction, F(4, 28) = 26.46, *p* < 0.001, post hoc tukey *p* < 0.01 in all passive versus active comparisons, as well as the drift duration, repeated-measures ANOVA with Greenhouse–Geisser correction, F(4, 28) = 21.67, *p* < 0.001, post hoc tukey *p* < 0.05 in all passive versus active comparisons, were smaller in the passive tasks compared with the active tasks. Although the saccade rate was lower in the two active tasks, the overall area encompassed by the FEM measured by ISOA was larger in the active tasks compared with the passive tasks, repeated-measures ANOVA with, F(4, 28) = 13.57, *p* < 0.001, post hoc tukey *p* < 0.001 in all passive vs active comparisons.

**Figure 3.**
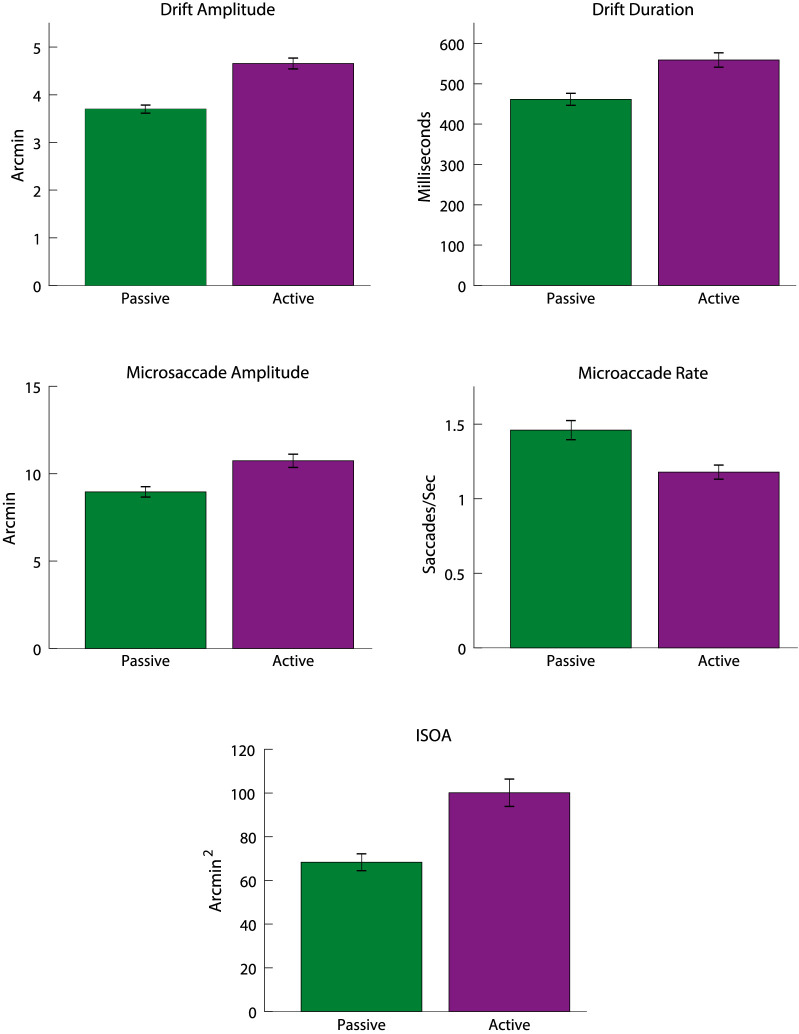
Comparison of FEM with the passive and active tasks pooled together. Error bars represent the standard error of the mean across all subjects for the combined conditions. Overall there were fewer, but larger, microsaccades in the active tasks compared to the passive. There were also larger and longer drifts in the active tasks. This led to an overall higher fixation area in active tasks as measured by the ISOA.

As can be seen in Figure 2, the FEM between the two passive tasks (Maltese cross and disk) were statistically similar as well as between the two active tasks (Vernier and tumbling E) (post hoc Tukey, *p* > 0.05 in all cases, with a single exception in drift duration between the two active tasks). So, to highlight the comparisons between passive and active fixation tasks, the tasks in each category were combined. The concentric circles condition did not show consistently significant differences from either task and so it was ignored for this analysis. Once combined, the difference between active and passive tasks were clear for all FEM metrics (paired-samples *t* test, *p* < 0.001). Figure 3 plots the combined and averaged data. The differences between tasks are more readily evident when the active and passive tasks are pooled together.

**Figure 4.**
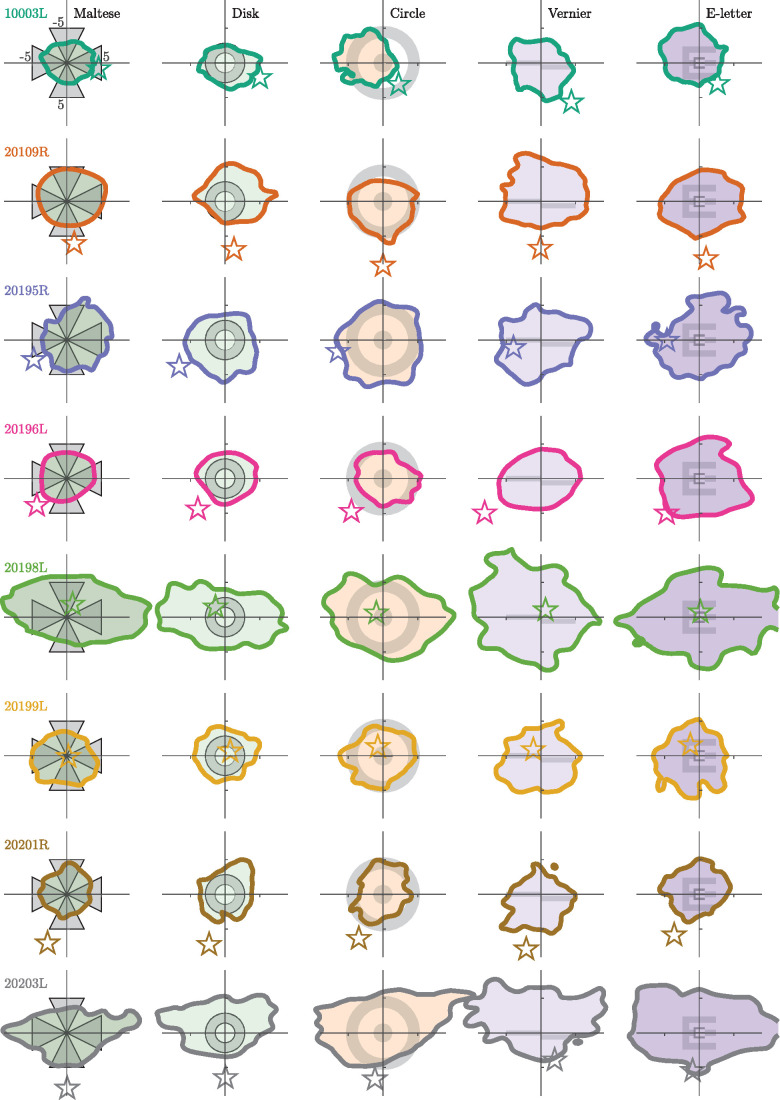
The 68% isoline contours (ISOA) of the entire eye movement trace. Each row is a subject and each column is a fixation condition, the stimulus for which is drawn centered on each plot for reference. Position (0,0) on the plot corresponds with the PRL, or the peak of the fixation position distribution. Note that idiosyncrasies in the eye movement can cause this peak to appear displaced from the center of the isoline contour. The position of these distributions represent the location of the fixated image and are plotted in fundus view coordinates (same as Figure 1). The star in each plot indicates the relative location of the PCD. Axis units in the upper left plot are in minutes of arc.

Figure 4 shows the 68% isoline contours for each subject for each condition centered on the PRL, which is defined at the peak of the fixation positions’ PDF. The stimulus for each condition is drawn in the center of each graph for reference, but the stimulus will sweep across the retina based on the extent of the eye movement. The overall fixation area was larger for the active tasks compared with the passive tasks (paired-samples *t* test, *p* < 0.001). Extensive intersubject variability is readily apparent in Figure 2. The average standard deviation of the ISOA between subjects for each condition (columns in Figure 4) was 43.53 arcmin${}^{2}$, whereas the average SD between conditions for each subject (rows in Figure 4) was 21.26 arcmin${}^{2}$. Although intersubject variability is extensive (over double the size of the difference between conditions), there remains a significant difference in fixation behavior between the five conditions. Large differences in fixation behavior between subjects is expected in measurements of FEM, especially when psychophysical expertise is taken into account (Cherici et al., 2012).

**Figure 5.**
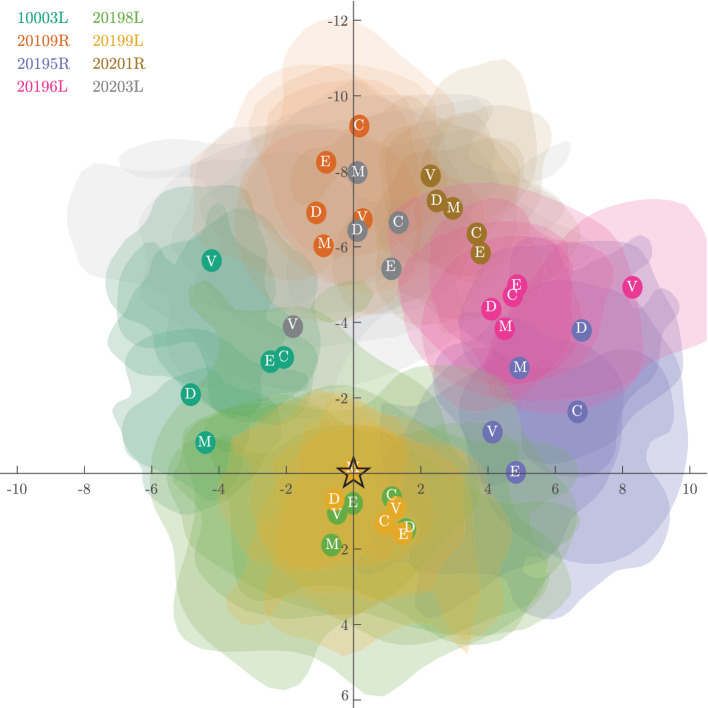
Individual PRL locations plotted relative to their PCD centered to (0,0). Location of the PRLs are shown as opaque circles overlaid onto the transparent isoline contours with the condition defined in white text within the center. This figure highlights that each subject's PRL tends to fall off their respective PCDs. However, the various PRLs defined in each condition all group together closely. To enhance visibility, the isoline contours here encompass only 38% (0.5 SD) of the fixation trace instead of the 68% (1 SD). Axis units are in minutes of arc.

Figure 5 shows the same data as shown in Figure 4, but in this case the ISOAs from all subjects and conditions are overlaid on a single plot with all subjects’ respective PCDs at (0,0). This figure reveals several phenomena. First, the PRL rarely coincides with the PCD and is displaced, on average by 5.20 arcmin (SD = 2.54 across tasks and between subjects, SD = 0.23 across subjects and between tasks). This is largely in line with other reports that the PRL does not perfectly correspond with the PCD (Li et al., 2010; Putnam et al., 2005; Wang et al., 2019; Wilk et al., 2017). Second, the PRL tends to be displaced above the PCD in fundus coordinates. This is consistent with recently published reports (Reiniger et al., 2021). Finally, subjects adopt a consistent PRL regardless of the task and its visual demand. The Euclidean distance between the PRL and the PCD did not significantly differ from one another (repeated-measures ANOVA with Greenhouse-Geisser correction, F(2.155, 15.087) = 0.313, *p* = 0.751). A Kolmogorov–Smirnov test for 2D distributions was used to determine whether eye position distributions, and therefore the PRL, differed between the different conditions. There was no difference in the PRL location between conditions for any subject (*p* < 0.001 in each of the five conditions and eight subjects). This finding is in agreement and extends on the finding that the PRL for a static Maltese cross target remains stable between days (Kilpeläinen et al., 2020).

**Figure 6.**
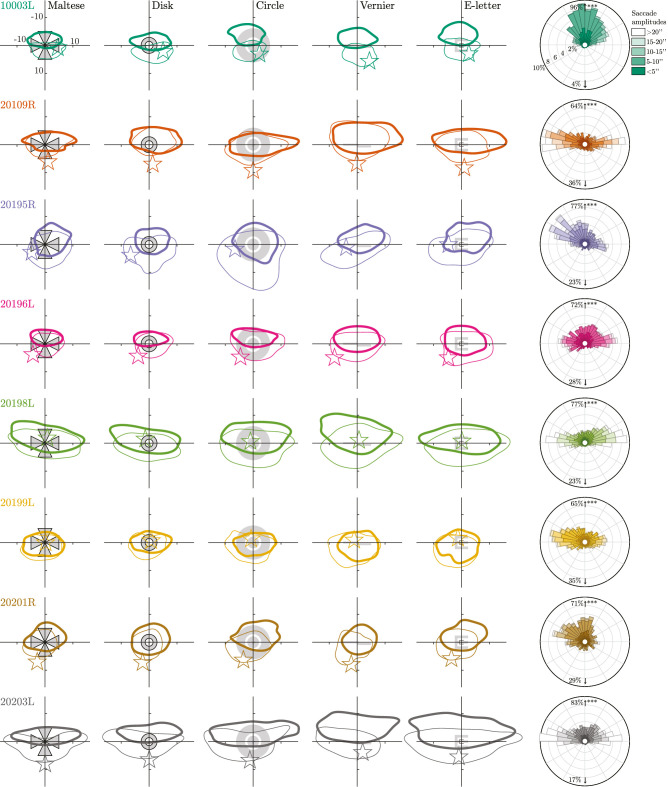
The 68% isoline contours for the location of the fixated image at the start and end of saccades, drawn as thin and thick contours, respectively. Each row is a subject and each column is a fixation condition, the stimulus for which is also drawn on the figure. Position (0,0) on the plot corresponds to the PRL location from Figure 4. The position of these distributions represent the location of the fixated image and are plotted in fundus view coordinates (same as Figure 1). The star indicates the location of the PCD. Axis units in the upper left plot are in minutes of arc. The right column contains rose plots indicating the percentage of microsaccades as a function of direction for each subject. Each petal in the plot is further broken down by saccade length. The percentage values written in the upper and lower fields indicate the total proportions of microsaccades with an upward vs a downward component, respectively. All subjects have a significant tendency for an upward component (see text).

Figure 6 shows two analyses of microsaccades. The left matrix of plots are 68% isoline contours for saccade start and end points, represented by thin and thick contours respectively. The data for each subject are further distilled into the rose plots on the right which aggregate the microsaccade data from all conditions. Some clear and distinct patterns emerge here. First, the ISOA for saccade start and end points cover a larger area than the conventional ISOAs of Figure 4, which encompass both saccade and drift periods. Second, the distribution of microsaccades tends to be more frequent and of larger amplitude in the horizontal direction, which is largely in agreement with other research (Cherici et al., 2012; Sheehy et al., 2020; Thaler et al., 2013). Finally, despite the more extensive horizontal spread, every subject shows a tendency to make saccades, on average, with an upward component (binomial test, looking for a proportion of 50%, *p* < 0.001 for all subjects). The saccades move the image upward on the fundus, straddling either side of the PRL. If the image moves up during a saccade, this means that the fovea moves down relative to it. In gaze coordinates, this corresponds with a saccade that redirects the gaze upward as the coordinates between fundus view and gaze coordinates are inverted. This movement could be classified as a form of spontaneous upbeat micronystagmus (Eggers et al., 2019), although in these instances, the upbeat nystagmus clearly does not indicate a pathological condition. A similar behavior is reported in other articles (Mestre et al., 2021; Stevenson et al., 2016), but not observed universally.

## Discussion

Our study shows large and significant variations of microsaccade and drift kinematics between subjects and between different tasks. We have confirmed that an individual's FEM behavior depends on the task involved. Specifically, we have confirmed that active tasks result in less frequent microsaccades (Bridgeman & Palca, 1980; Martinez-Conde et al., 2004) giving rise to correspondingly longer and larger drift epochs which, in turn, cause the microsaccades to be larger. This pattern of behavior leads to overall larger ISOAs during active tasks. Passive tasks, by comparison, are marked by shorter and more frequent microsaccades and smaller, briefer drift epochs, all leading to a smaller ISOA. The increase in ISOA from passive to active tasks was 57 ± 23 % on average. These results are consistent with the notion that FEM constitute a behavior that is subconsciously mediated to serve different functions depending on the task at hand. Indeed, evidence of reduced microsaccade rate and a seemingly optimal control of drift eye movements was recently reported for subjects asked to read a line on a Snellen acuity chart (Intoy & Rucci, 2020). The exact extent that FEM may be modulated to enhance vision is still unclear and a matter of ongoing experimentation (Kagan et al., 2008; Rucci et al., 2018). During the active tasks subjects tended to suppress their microsaccades because the rapid transients from these movements can be detrimental to fine-scale discrimination due to microsaccadic suppression (Bridgeman & Palca, 1980; Intoy et al., 2021), either from blurring of the retinal image or central suppression.

The consistency of the PRL location between tasks was tested using a 2D Kolmogorov–Smirnov test and was found to remain the same regardless of the fixation target. This extends on a recent report that the PRL for a Maltese cross target does not change between hours and across days (Kilpeläinen et al., 2020). However, the possible shifts in the location of the PRL have not been investigated for binocular viewing conditions or for more complex viewing experiences, such as during smooth pursuit or fixation within extended scenes.

Although the PCD on the retina offers the best location for photoreceptor spatial sampling (according to the Nyquist sampling limit), the PRL rarely aligns with it exactly. Previous research has consistently shown the same (Putnam et al., 2005; Wang et al., 2019; Wilk et al., 2017). We found the average separation between the PCD and the PRL to be 5.20 arcmin. In the majority of cases the PRL is positioned superior to the PCD (in fundus view coordinates). This reflects a similar tendency for the PRL in individuals with a central scotoma to adopt an eccentric PRL in the superior retina (Messias et al., 2007; Verdina et al., 2017). This means that the retinal location with the PCD is sampling a part of the visual field just above the direction of gaze. This tendency has been reported previously by another group (Reiniger et al., 2021), who also used an AOSLO. These displacements are very small and in our opinion, as discussed in one of our previous articles (Wang et al., 2019), seem unlikely to have any functional importance.

A subclinical form of upbeat nystagmus was present to varying extents in all of our subjects (see Figure 6 right column). Similar behavior was reported for some, but not all of the subjects in two other studies (Mestre et al., 2021; Stevenson et al., 2016) and is further evident in a slight upward tendency (although not commented on) in the saccade distribution plots of other papers (Cherici et al., 2012; Thaler et al., 2013). Interestingly, these saccades had the tendency to direct the gaze above the PRL, with the following drift generally bringing the gaze downward toward the PRL. This pattern was present in all subjects and suggests that, when classifying the PRL, it is important to consider the complimentary relationship between drifts and saccades. More work is necessary to assess the PRL overshoot behavior and complementary behavior of the following drift segment. In any case, the minutiae of FEM reveals that a PRL that is identified by any of the current methods, including the ISOA approach used here, may be ill-defined. This topic is part of an ongoing investigation.

Although we measured significant and informative differences in FEM between conditions, we found that differences in FEM between individuals are even greater. The standard deviation of the ISOA between individuals, for example, was roughly twice that between conditions. These differences can be partly explained by experience (Cherici et al., 2012). All of our subjects were recruited from within the UC Berkeley School of Optometry community and therefore had some experience sitting for visual psychophysics experiments and/or for clinical examinations. However subjects 10003, 20109, and 20196, who all had ISOAs that were lower than the mean, have logged dozens of hours in psychophysics experiments related to eye tracking, including AOSLO psychophysics experiments.

When considering FEMs, it is prudent to consider the goals for maintaining a subject's steady fixation, because all fixations are not equal. If the goal of the fixation target is to minimize the overall movement of the eyes (as is the case in many clinical situations), then one must consider which types of FEM are most likely to be an impediment. If the rapid transients from saccades are most likely to have a deleterious effect then it is preferred to rely on an active task so the subject will suppress their microsaccades in order to perform the task. If the goal is to minimize the total area covered by the fixation, then choosing a more passive fixation task is likely to be most effective. Of course, given the effect of intersubject variability seen in these data, as well as other studies, it is also prudent to keep in mind that subject instructions and recruitment play a large role in the stability of fixation as well.

## Conclusion

This study examined the influence of different fixation targets and tasks on FEM and the location of the PRL in healthy eyes. Using an AOSLO, we developed a new method to locate and follow the target projected on the retina over time relative to the PCD. We confirmed the non-normality of the eye motion distribution, hence the necessity to rely on better descriptors of fixation stability indices such as ISOA and its accuracy to estimate each individual's PRL. The different fixation tasks consisted of active tasks, which had temporal variation and required subject responses, and passive tasks, where the subjects were instructed to simply hold their gaze on the target. The active tasks elicited larger but fewer microsaccades. Consequently, the amplitude and duration of intersaccadic drifts were significantly larger. Larger and longer drifts combined with larger microsaccades led to larger overall fixation instability, as quantified by the ISOA. Our result suggests that subjects suppress their microsaccades during active tasks, and the subsequent longer drift epochs would cause the object to move away from the PRL, thereby requiring a relatively larger microsaccade to reorient. Finally, although the FEM were significantly modulated by the task, the intersubject variability was expectantly substantial. The two to four times larger effect on fixation stability across individuals compared to task suggest that experimenters might, when aiming to better control the user's eye position, put a greater emphasis on instructions, training, and subject recruitment rather than on the fixation stimulus itself.

## Supplementary Material

Supplement 1

Supplement 2

## Supplementary materials

### Example movie

The attached movie shows on the left how the AOSLO raster appears to the subject for the concentric circles task. The right side shown the simultaneously recorded video of the retina of subject 20201R. Three seconds out of a full 36-second video is shown. The movement of the retina arising from fixational eye motion and its amplitude relative to the size of the stimulus is readily evident. This segment also highlights how the decrement stimulus is inscribed into the video file directly, providing a completely unambiguous record of the stimulus’ position across the photoreceptor mosaic. Note that this segment has been compressed to keep the file size small and does not represent the raw videos used in stabilization and analysis.

### Instructions

The instructions given to each subject were identical from one subject to the next. This script was read aloud to each subject for each condition and any clarifying questions were answered before the experiment began. Note that experimenters asked the subject to blink when their tear film degraded to the point of interfering with the video quality.

#### Maltese

In this experiment you will be required to look toward the center of the cross for the entire duration of the 36 second video. There will be a sound played when the video starts and stops recording. You are allowed to blink as needed and I will ask you to blink if necessary. Press the start button when you are ready to go.

#### Disc

In this experiment you will be required to look towards the center of the disc for the entire duration of the 36 second video. There will be a sound played when the video starts and stops recording. You are allowed to blink as needed and I will ask you to blink if necessary. Press the start button when you are ready to go.

#### Concentric circles

In this experiment, you will be required to look towards the center of the target for the entire duration of the 36-second video. You will see a series of rings that become smaller. There will be a sound played when the video starts and stops recording. You are allowed to blink as needed and I will ask you to blink if necessary. Press the start button when you are ready to go.

#### Vernier

In this experiment, you will be required to look at the two lines for the entire duration of the 36 second video. The two lines will vary in position at random intervals which will be announced by an audible cue. Using the up and down buttons, report if the right line is higher or lower than the left line. There will be a sound played when the video starts and stops recording. You are allowed to blink as needed and I will ask you to blink if necessary. Press the start button when you are ready to go.

#### Tumbling E

In this experiment you will be required to look at the “E” for the entire duration of the 36 second video. The letter “E” will change size and direction at random intervals which will be announced by an audible cue. Using the buttons, report the direction the “E” is facing: up, down, left, or right. There will be a sound played when the video starts and stops recording. You are allowed to blink as needed and I will ask you to blink if necessary. Press the start button when you are ready to go.

### Extra tables

The attached .csv files contain the raw data for each of the five eye motion parameters shown in Figures 2 and 3. Rows indicate different subjects and columns indicate different conditions. The variable names and related units are shown in the top, left of each table.
